# Predicting suicide attempts among Norwegian adolescents without using suicide-related items: a machine learning approach

**DOI:** 10.3389/fpsyt.2023.1216791

**Published:** 2023-09-26

**Authors:** E. F. Haghish, Nikolai O. Czajkowski, Tilmann von Soest

**Affiliations:** ^1^Department of Psychology, Faculty of Social Sciences, University of Oslo, Oslo, Norway; ^2^Department of Mental Disorders, Division of Mental and Physical Health, Norwegian Institute of Public Health (NIPH), Oslo, Norway; ^3^Norwegian Social Research (NOVA), Oslo Metropolitan University, Oslo, Norway

**Keywords:** suicide attempt classification, risk factors, supervised machine learning, adolescents, survey

## Abstract

**Introduction:**

Research on the classification models of suicide attempts has predominantly depended on the collection of sensitive data related to suicide. Gathering this type of information at the population level can be challenging, especially when it pertains to adolescents. We addressed two main objectives: (1) the feasibility of classifying adolescents at high risk of attempting suicide without relying on specific suicide-related survey items such as history of suicide attempts, suicide plan, or suicide ideation, and (2) identifying the most important predictors of suicide attempts among adolescents.

**Methods:**

Nationwide survey data from 173,664 Norwegian adolescents (ages 13–18) were utilized to train a binary classification model, using 169 questionnaire items. The Extreme Gradient Boosting (XGBoost) algorithm was fine-tuned to classify adolescent suicide attempts, and the most important predictors were identified.

**Results:**

XGBoost achieved a sensitivity of 77% with a specificity of 90%, and an AUC of 92.1% and an AUPRC of 47.1%. A coherent set of predictors in the domains of internalizing problems, substance use, interpersonal relationships, and victimization were pinpointed as the most important items related to recent suicide attempts.

**Conclusion:**

This study underscores the potential of machine learning for screening adolescent suicide attempts on a population scale without requiring sensitive suicide-related survey items. Future research investigating the etiology of suicidal behavior may direct particular attention to internalizing problems, interpersonal relationships, victimization, and substance use.

## Introduction

Suicide attempt is defined as engaging in self-harm behavior with at least some intention to die (1) and its prevalence during adolescence is estimated to be up to 10% with a peak around the age of 16 (2). Cross-national studies also emphasize the high prevalence of suicide attempts during adolescence and early adulthood (1, 3). Thus, identifying adolescents at risk of attempting suicide is a key step toward offering early interventions and mitigating its substantial public health impact. Yet, identifying at-risk adolescents is a challenging task, because suicide attempt risk is tied to a variety of individual, societal, cultural, and environmental factors (4) and despite decades of extensive research, the predictive power of identified risk factors remains weak and inconsistent (5). A systematic meta-analysis on clinical instruments revealed a low combined positive predictive value of 5.5% for suicidal behavior, concluding that the “high-risk” classification was not clinically useful (6). Adolescents’ suicide attempts can also be unpremeditated or compulsive, making risk estimation even more difficult (7–9). As a result, recent reviews noted that prediction of suicidal behavior needs to account for the complex interplay of a multitude of risk factors, shifting the focus from risk factors to risk algorithms (5, 10).

Not only is the prediction of suicide attempt risk a challenging endeavor, but the sensitive subject matter also poses difficulties for assessment in the general population (11–13). Therefore, suicidal behavior is often not measured in population-based studies (12, 14, 15). Research has shown that prevalence of self-reported suicidal behavior in anonymous surveys is up to three times higher than non-anonymous surveys, further stressing sensitivity of suicide-related items (16, 17). Therefore, recent studies have stressed the importance of identifying individuals at risk for suicide attempts when suicide-related items, such as suicidal ideating and self-harm behavior are not assessed (18).

Recently, the potential of machine learning techniques to improve suicide attempt classification has been explored. Machine learning algorithms can make use of a multitude of variables and evaluate their complex interplay, resulting in promising improvements in the field of suicide research (19–21). However, these studies have focused on variables directly related to suicide, such as suicidal ideation and self-harm behaviors, and consistently identified them as the strongest predictors (19, 22–26). Moreover, there seems to be insufficient focus on the practicality of implementing machine learning algorithms at a population level, especially when suicide-related data are lacking. Although machine learning is regarded as a promising tool for predicting suicide attempts, a strong focus on suicide-related data may serve as a barrier to its implementation at scale. As a result, less is known about the performance of machine learning models in predicting suicide attempts when suicide-related items are not included (18).

### The current study

In this study, we aim to answer two research questions. First, how accurately can machine learning models identify adolescents who self-report a recent suicide attempt without using sensitive suicide-related information such as self-harm, suicide plan, suicide ideation, or history of previous suicide attempts? Second, what are the most important personal, psychological, societal, and environmental variables associated with adolescents’ recent suicide attempts? As noted in the literature, the risk factors of suicide attempts are usually studied in isolation and it is not clear what the most important predictors are, once hundreds of potential predictors are being taken into consideration (5, 10). Due to the low prevalence of suicidal behavior, richness and size of data is central to studying suicidal behavior with machine learning, both for classifying suicide attempt accurately as well as evaluating the predictors’ importance; however, previous studies of machine learning frequently suffer from either small sample sizes or using non-representative samples (23–29). We use nationally representative retrospective cross-sectional survey data from 173,664 Norwegian adolescents and analyze a multitude of items from a variety of psychological, sociological, and environmental domains to address our research questions.

## Methods

### Sample and procedure

This study utilizes the Ungdata surveys, a data collection scheme developed to administer youth surveys at municipal levels in Norway, assessing junior and senior high school students (grades 8–13, ages 13–18). The data were collected between 2014 and 2019 across most of municipalities in Norway, with students being invited to complete an electronic questionnaire in their classrooms. The current data analysis was performed on a subset of dataset, which included 169 items that were received by all participants (*n* = 173,664).

### Measures

The analysis included 169 survey items. The full description and response options of these items can be found on the OSF repository of the study via https://osf.io/2qfnc/.

#### Recent suicide attempt

The outcome variable was recent suicide attempts, which was measured with a single self-report item, asking “*Have you tried to take your own life in the last 12 months?*,” with the response options “Yes” and “No.”

#### Socio-demographics

In addition to participants’ gender and age, various socio-demographic factors were evaluated. These included socio-economic status, assessed using the second edition of the Family Affluence Scale (30, 31), parents’ education level, adolescents’ subjective assessment of their financial status in recent years, and the quality of their local environment. Participants also responded to items about future expectations such as higher education, career, and life quality, reflecting on their aspirations.

#### Interpersonal relationships

Interpersonal relationships were assessed through a comprehensive set of items inquiring about sexual and romantic relationships, relationships with parents, relationships with teachers and peers, and activities on social media. In addition, adolescents were asked to report online friendships and their parents’ supervision of their social media activities online.

#### Somatic health, mental health, and victimization

Several items measured somatic health, physical pain, and hospitalizations, along with other health aspects such as exercising habits and dietary practices. For example, participants reflected on their consumption of vegetables, fruits, junk food, and energy drinks, adherence to strict diets, and concerns about body weight. Several mental health instruments were also administered, including the measurement of depressive and anxiety symptoms using a short version of the Hopkins Symptom Checklist (32). Self-esteem was assessed with items from the Self-Perception Profile for Adolescents (33), and loneliness was assessed using the short form of the revised UCLA Loneliness Scale (34). In addition to somatic and mental health evaluations, participants’ exposure to victimization, including bullying and physical, verbal, or sexual victimization in settings such as family, school, or social media, was assessed (35).

#### Conduct problems and substance use

Conduct problems were assessed through a series of questions drawn from Olweus’ scale of antisocial behavior (36) and the National Longitudinal Youth Survey (37). Participants were also queried about their use of substances such as alcohol, tobacco, snus (an oral tobacco product), cannabis, and other illegal substances. Additionally, the alcohol consumption of parents and friends was assessed, along with the respondents’ perception of alcohol consumption norms within their family.

#### Other measures

Various other aspects of adolescents’ lives were also assessed, including leisure activities, lifestyle, online and offline activities, participation in different youth clubs and organizations, individual values, and political attitudes.

### Statistical analysis

The data were prepared and analyzed using R statistical software version 4.1 (38) and all the machine learning models were built using the h2o R package version 3.30.0.6 (39).

#### Procedure

To predict recent suicide attempts among adolescents, the Extreme Gradient Boosting (XGBoost; (40)) algorithm was examined, which is based on boosting decision-tree techniques. The algorithm was trained using 80% of total data, reserving 20% of the sample for testing. Instead of a random split, stratified random splitting was employed from the splitTools R package (41) to ensure the prevalence of suicide attempts remained consistent in both the training and testing datasets.

#### Class imbalance

The prevalence of suicide in the population is low, creating a severe imbalance between the classes (suicidal vs. non-suicidal), which can bias both the model and the measures used to evaluate its performance in favor of the majority class (42–44). A standard solution is to artificially balance the training dataset by either up-sampling the minority class or down-sampling the majority class. However, modifying the underlying distribution of the outcome breaches the principal machine learning assumption that the training and testing datasets are sampled from the same population (45). To resolve this issue, previous machine learning studies on suicide attempt prediction have often balanced the testing dataset as well [see for example, (26, 29)], rendering the model inapplicable to the real-world problem. Simply put, when the distributions of both the training and testing datasets differ from the underlying population distribution, the performance of the model on a real-world dataset with severe class imbalance would be unknown (45).

Another approach suggests preserving the testing dataset to represent the real-world distribution (46) and calibrating the trained model’s probabilities before evaluating its performance on the testing dataset (45, 47, 48). This method addresses the imbalance problem while keeping the model pertinent to the actual outcome distribution. In accordance with this strategy, we balanced the training dataset by up-sampling the minority class through bootstrapping, calibrated the models’ probabilities using monotonic transformation, provided by the h2o R package, and then assessed the performance of the selected models on the testing dataset.

#### Fine-tuning and model evaluation

To fine-tune the models, random search was employed (49) and early stopping strategies were implemented during the search to mitigate overfitting (50). Each model was assessed through 10-fold cross-validation and optimized and ranked according to the Area Under the Precision-Recall Curve (AUPRC). Traditionally, the Area Under the Curve (AUC) of the Receiver Operating Characteristic (ROC) curve is used to optimize and evaluate binary classifiers (51). Under severe class imbalance, however, AUC can be overly optimistic, making AUPRC the preferred measure for assessing the model’s performance (52). Yet, to make our results comparable to other suicide prediction studies, we continue to report AUC alongside AUPRC. When the random search process stopped, the model with the highest AUPRC (on the training dataset) was selected and further examined with the unseen testing dataset, and metrics such as AUC, AUPRC, sensitivity, and specificity were computed and reported.

#### Predictor importance

To evaluate the predictors’ importance, we employed SHapley Additive exPlanations [SHAP; (53)] method, which is inspired by Shapley values in cooperative game theory (54, 55). Initially, we will present a SHAP summary plot to depict the contribution of each variable across all subjects (rows in the dataset) within the test dataset. This procedure differs from determining predictor importance based on the gain of the loss function during the construction of decision trees. The advantage of this method is that it offers detailed insights into the importance of each variable throughout its entire range of values. Hence, SHAP also enhances the transparency of the model by illustrating how different values of each item influence the model’s decision-making. We identify the top 15 predictors from a total of 168 items included in the model, ranking them according to the normalized mean absolute SHAP value in descending order [see (56, 57)]. By normalizing the mean absolute SHAP value, ranging from 0 to 1, we can compare the relative importance of the top predictors. It should be noted that the term “predictor” may be contentious, as we are utilizing retrospective cross-sectional data. In this context, we use “predictor” solely to refer to a survey item that provides the model with unique information, facilitating the evaluation of suicide attempt risk (i.e., the likelihood that an adolescent has attempted suicide within the past 12 months) and classification, without implying any causal relationship between the item and the outcome.

#### Missing data imputation

The average missing rates of the data was 2.64%. The regularized iterative Factorial Analysis for Mixed Data (FAMD) algorithm from the missMDA R package (58) was utilized to conduct a single imputation. This algorithm flexibly imputes numerical and factor variables (59, 60) and, importantly, offers a fast and scalable imputation solution for large datasets. Except for the descriptive statistics, all other analyses were carried out on the imputed dataset.

**Figure 1:**
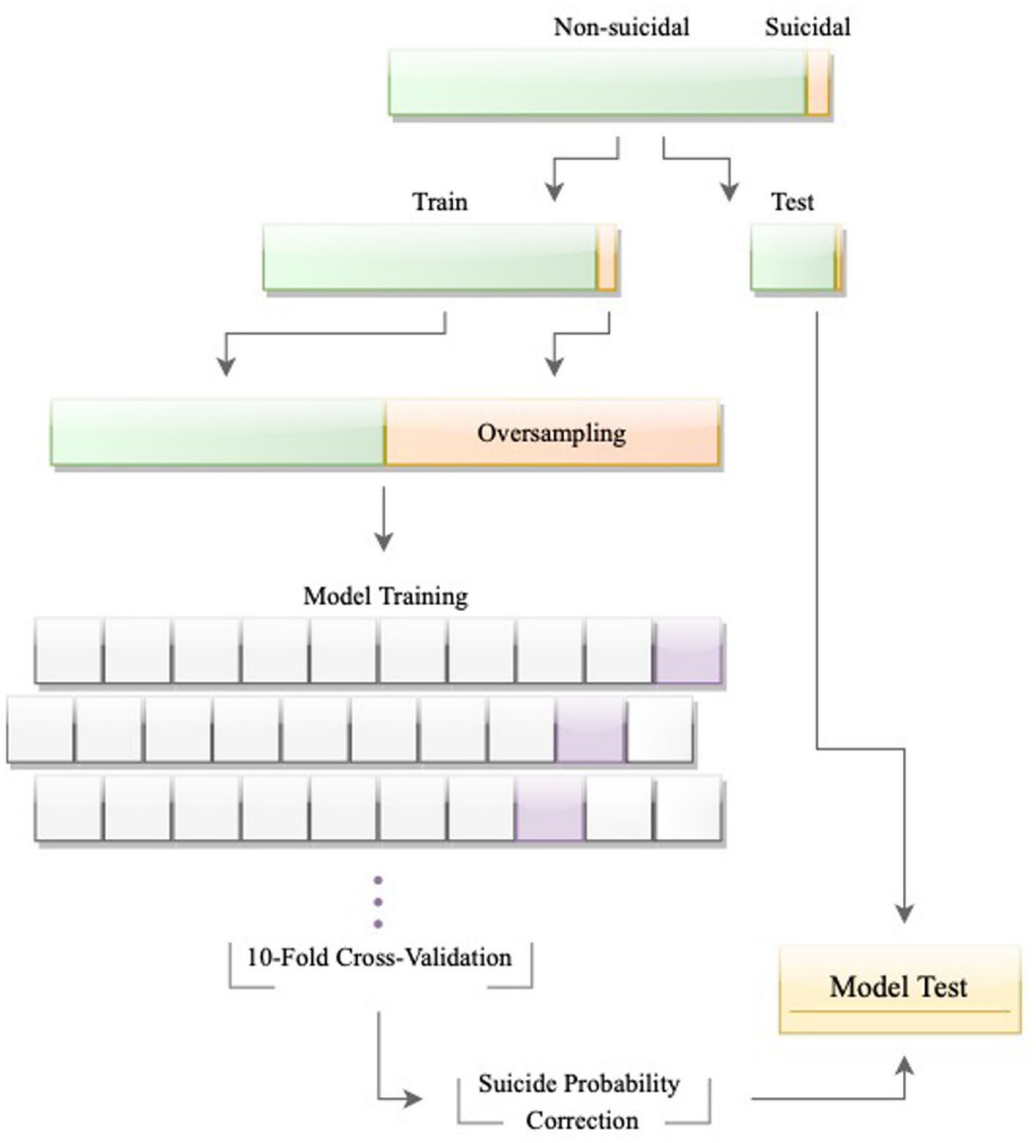
The process of model development and evaluation.

## Results

The prevalence of self-reported recent suicide attempt in the sample was 4.66% (*n* = 8,090), with a male prevalence of 3.37% (*n* = 2,824) and a female prevalence of 5.87% (*n* = 5,040). There were 2.28% missing data on gender, and a Chi-square test revealed a significant difference between genders [*χ*^2^ (1) = 579.52, *p* < 0.001]. According to Table 1, students in their third year of junior high school (age 15) and first year of senior high school (age 16) reported the highest prevalence of self-reported recent suicide attempts, both at a rate of 5.21%. Conversely, the lowest rate was observed among third-year senior high school students, at a rate of 3.20%. Students who reported no parental higher education had higher suicide attempt rates compared to others, as detailed in Table 1. Additionally, the majority of adolescents who reported a recent suicide attempt had not met with a psychologist (56.22%) or visited a health clinic for adolescents (69.67%), a school nurse (42.89%), or an accident and emergency ward (51.42%) in the past 12 months.

**Table 1:** Characteristics of the high-risk and no-risk suicide groups in the raw dataset.

Items (missing rate)	Reported recent suicide attempt (*n* = 8,090)	No reported recent suicide attempt (*n* = 165,574)
Gender (2.28%) *		
Boy	2,824 (3.37%)	80,983 (96.63%)
Girl	5,040 (5.87%)	80,863 (94.13%)
School year (1.58%) *		
Junior (age 13)	1,391 (4.12%)	32,395 (95.88%)
Junior (age 14)	1,673 (5.06%)	31,389 (94.94%)
Junior (age 15)	1,735 (5.21%)	31,571 (94.79%)
Senior (age 16)	1,760 (5.21%)	32,004 (94.79%)
Senior (age 17)	1,013 (3.93%)	24,792 (96.07%)
Senior (age 18)	359 (3.20%)	10,845 (96.80%)
Father education (13.59%) *		
Higher education	3,967 (3.93%)	96,891 (96.07%)
No higher education	2,757 (5.60%)	46,450 (94.40%)
Mother education (11.79%) *		
Higher education	4,518 (3.96%)	109,450 (96.04%)
No Higher education	2,466 (6.29%)	36,758 (93.71%)
Subjective SES (2.14%) *		
Well-off	2,164 (2.90%)	72,415 (97.10%)
Average	5,211 (5.58%)	88,124 (94.42%)
Poor	474 (23.37%)	1,554 (76.63%)

*Chi-square test of independence is significant with *p* < 0.001.

Reported values are from the raw dataset, prior to the imputation.

### Model performance

Upon examination with the testing dataset, the best model achieved an AUPRC of 0.471 and an AUC of 0.921. The sensitivity and specificity of the model on the testing dataset were 0.77 and 0.90, respectively. This indicates that 77.0% of the adolescents who reported recent suicide attempt could be correctly classified, while simultaneously correctly classifying 90.0% of adolescents who did not report a recent suicide attempt. Despite the low prevalence of recent suicide attempts at the population level, this result indicates that the best-performing model could identify the majority of adolescents who reported a suicide attempt with high specificity.

### Predictor importance

To address our second research question, we investigated the importance of the 168 items that were entered in the model as potential predictors. Table 2 shows the scaled relative importance of the top 15 predictors of suicide attempts, which were computed by normalizing mean absolute SHAP values. Of these, 7 items were related to the domain of mental health, assessing symptoms of depression, anxiety, self-esteem, life satisfaction, or use of mental health service (e.g., “felt unhappy, sad, or depressed,” “felt worthless,” “satisfied with my appearance,” “felt scared for no reason,” and “contact with a psychologist/psychiatrist”). The item “felt unhappy, sad, or depressed” was the most important predictor of suicide attempt. In addition to mental health, items in the domain of victimization (e.g., “being teased or threatened”), substance use (“smoking” and “having been offered cannabis”), interpersonal problems (e.g., “satisfied with my parents,” “having nobody to share problems with”), and lastly, participants’ age were also among the most important predictors of suicide attempts. These prominent predictors, thus, were reflecting on four main domains of mental health, especially internalizing problems and substance use, victimization, and interpersonal relationships.

**Table 2:** Top 15 predictors of adolescents’ suicide attempt and their relative importance.

Items	Relative Importance
Felt unhappy, sad, or depressed	1.000
Contact with a psychologist/psychiatrist	0.621
Felt worthless	0.477
Disappointed with myself	0.393
Smoking	0.360
Satisfied with my appearance	0.338
Satisfied with my parents	0.319
Felt scared for no reason	0.309
Being teased or threatened	0.296
Talking to parents in case of a problem	0.239
Having nobody to share problems with	0.227
Felt hopelessness about the future	0.213
Having been offered cannabis	0.204
Age	0.203
Having a partner or reporting a breakup	0.203

Relative importance represents normalized means absolute SHAP value.

**Figure 2:**
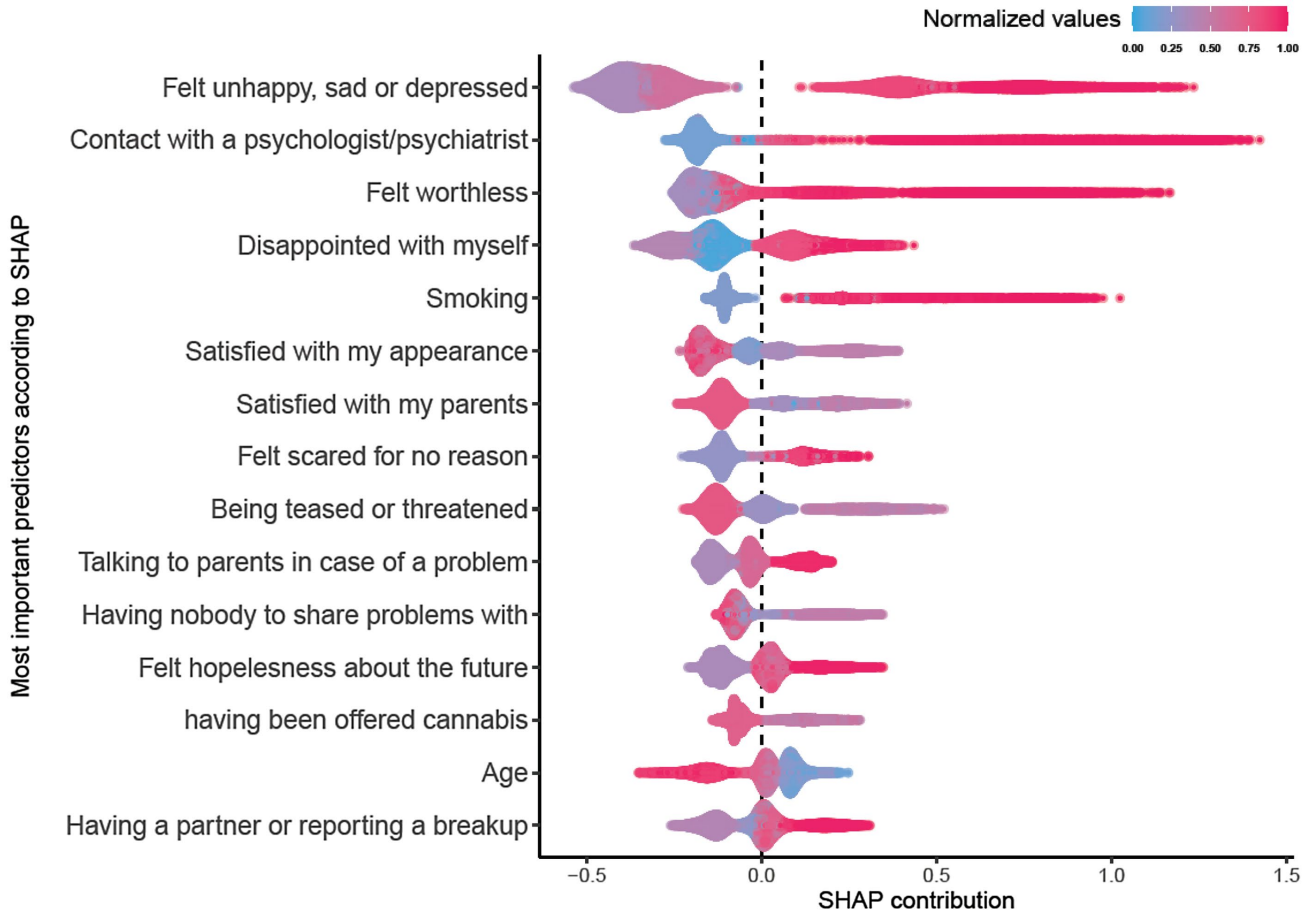
SHAP contribution of the top 15 predictors.

We visualized the SHAP values to elucidate how the significant predictors are related to the model’s output for all participants in the testing dataset. In Figure 2, each of the top 15 predictors is presented in a separate row, with color coding representing the normalized data point, ranging from blue (0) to red (1). Take, for example, the blue section of the “Smoking” item corresponds to low values such as “I’ve never smoked,” while red dots symbolize high values such as “I smoke every day.” In addition to the color range, Figure 2 also illustrates the SHAP contribution of each variable, which can be negative, positive, or zero. Positive SHAP values signify importance for positive classification and vice versa, while SHAP values close to zero denote unimportance. For example, adolescents who have had frequent contact with a psychologist or psychiatrist (thus a red color) have a high and positive SHAP values, indicating that this information supported the model to distinguish adolescents who had reported suicide attempts from other adolescents. Regardless of the direction of the SHAP values, the higher the absolute value, the more important the predictor. In Figure 2, for example, the item “felt unhappy, sad or depressed” has a high overall SHAP value at both negative and positive sides, indicating that this item strongly supported the model’s classification. Figure 2 also shows that while taking hundreds of items into account, having lower age (shown in blue color) is indicator of a higher suicide attempt risk compared to higher age (red color).

## Discussion

The aim of this study was to identify adolescents who had attempted suicide during the past 12 months without utilizing other sensitive suicide-related survey items as predictors. In doing so, we employed population-based national data to fine-tune the XGBoost machine learning algorithm based on a multitude of psychological, social, and environmental survey items. The fine-tuned model reached an excellent AUC of 92.1% (61). This figure is near the highest AUC reported in existing literature, ranging from 0.716 to 0.925 (23, 25, 26, 28). With a high specificity of 0.90 and a high sensitivity of 0.77, the model could correctly classify 77% of adolescents reporting a recent suicide attempt as well as 90% of adolescents not reporting a recent suicide attempt. Such performance is promising and indicates the feasibility of classifying adolescents’ suicide attempts at scale with substantial accuracy, yet without utilizing any suicide-related items. Consequently, our findings answer our first research question, providing substantial evidence that high classification accuracy for adolescents’ recent suicide attempts at the population scale can indeed be achieved without the need to rely on suicide-related items. This result has important implications for the broader field and could guide future efforts in developing scalable, sensitive, and effective tools for identifying and addressing suicide risk in young populations.

To address our second research question, we pinpointed the most important predictors and provisionally grouped them within four main domains of internalizing problems, interpersonal relationships, victimization, and substance use. Our findings are in line with existing literature showing that internalizing problems, particularly depressive symptoms, are key predictors of suicidal behavior among adolescents (7, 8, 62, 63). Similar to our findings, Carballo et al., (62) also identified conflicts in interpersonal relationships to be among the major risk factors of suicidality among adolescents [see also (64)]. Moreover, a review by Calati et al. (65) underscores the importance of interpersonal factors for suicidal outcomes. Reviews have also repeatedly emphasized the importance of childhood maltreatment and bullying experiences for understanding suicidal behavior (7, 9, 66, 67), thereby supporting the role of victimization experiences. Finally, substance use and more specifically, smoking, was also among the most important predictors of adolescents’ suicide attempt. This aligns with numerous studies that recognize smoking as a general risk factor, independent of other socio-demographic variables (68–71), although the association between smoking and suicide has been debated (72). Our analysis accentuates that frequent smoking is indeed related to adolescent suicide attempts, even when accounting for hundreds of other items.

In our analysis, while focusing on the important predictors of suicide attempts, we also observed that certain mental health-related factors were not as influential in the model as might have been expected based on prior literature. This warrants a closer examination of these seemingly inconsistent findings. For example, looking at Tables 1 and 2, despite significant variations in the suicide attempt rate across socio-demographic variables such as age, gender, and socio-economic status, only age emerged as one of the important predictors. Moreover, among the top 15 predictors, age contributed among the least in terms of SHAP values. The fact that gender was not identified as an important predictor is particularly noteworthy. This contrasts with existing literature, including findings by Esang and Ahmed (69), where gender has been recognized as a significant factor. Given the marked prevalence differences between boys and girls in our dataset, the lack of gender’s role in the model requires careful consideration. A plausible explanation for this inconsistency may lie in the complex interplay of internalizing problems, interpersonal problems, and victimization experiences. These domains may subsume much of the demographic differences related to suicide attempts, rendering gender a comparatively less important predictor. Finally, in line with the existing literature, externalizing problems and delinquency did not emerge among the most important predictors of adolescent suicide attempts (69).

### Limitations and strengths

Several limitations of the present study must be mentioned. Most notably, the assessment of suicide attempts through a single self-report item lacks detailed information about the nature, seriousness, and context of the attempt (73). However, as noted at the outset of the paper, collecting such sensitive items in nation-wide surveys from hundreds of thousands of adolescents can be difficult, in contrast to clinical samples or smaller population-based samples. Yet, despite the absence of detailed data to distinguish between severe and less severe suicide attempts, the high performance and generalizability of our model provide assurance that the accurate identification of adolescents who have self-reported a recent suicide attempt is feasible, a finding of considerable significance. Additionally, our study employs retrospective cross-sectional data, limiting our understanding of the temporal development or severity of the predictors. While we have identified key psychological, social, and environmental predictors through comprehensive item analysis, the cross-sectional nature of our data precludes any inference about causality between these predictors and suicide attempts. However, as mentioned above, it is worth noting that our findings align well with existing literature that has established similar results in longitudinal studies. Emerging evidence suggests that risk scores derived from machine learning binary classification models using retrospective data can accurately predict future suicide attempts, including for those attempting for the first time [see (74, 75)]. Interestingly, these estimated risk scores also appear to be indicative of the severity of the suicide attempt. Recent longitudinal work demonstrates that higher estimated risk scores are inversely associated with the likelihood that adolescents will inconsistently report a prior lifetime suicide attempt in a 2-year follow-up assessment (76). These findings indicate that although our model is trained with retrospective cross-sectional data and cannot clarify causal relationships, yet, it provides real-world applications for identifying adolescents at risk of suicide attempts. Finally, we did not evaluate the model fairness, an issue increasingly emphasized in contemporary computer science literature. Warnings have been issued that even highly accurate models may underperform for minority groups, thereby perpetuating systematic inequality in fields such as social and health sciences (77). Future studies should aim to ascertain that models do not discriminate against specific socio-demographic groups, particularly if the model is intended for practical application (78).

Our paper also has a few significant strengths. The large sample size is particularly advantageous, as the low prevalence of suicide attempts necessitates a substantial number of participants to train robust classification models effectively. Additionally, the extensive array of survey items enables a comprehensive analysis, encompassing a myriad of potential risk and protective factors. This is particularly crucial given the complex nature of suicidal behavior (79), allowing the machine learning algorithms to account for intricate interactions between variables to refine predictive accuracy. Methodologically, we employed rigorous techniques to manage class imbalance and to evaluate and rank key predictors using a model-agnostic approach.

## Conclusion

The utility of models requiring suicide-specific information for risk estimation of suicide attempts is questionable because such data are challenging to collect on a population scale, especially from adolescents. Our study demonstrates that it is possible to classify adolescents reporting recent suicide attempts with high accuracy without depending on such sensitive items. A novel contribution of our study is the identification of key factors—namely, internalizing problems, interpersonal relationships, victimization, and substance use—as important predictors of suicide attempts in this population, while accounting for a myriad of other items across different domains. A focus on these aspects is warranted when identifying at-risk adolescents, particularly from longitudinal research, which can offer invaluable insights into the causal dynamics between identified factors and adolescence suicide attempts.

Future research should address the limitations of our current study and align its findings with existing theoretical frameworks on suicidal behavior. Subsequent investigations should also examine the latent factorial structure of these predictors and consider whether different machine learning algorithms would identify similar important items. There is also an opportunity to explore the use of stacked ensemble meta-learners, constructed by combining multiple machine learning models, for achieving superior classification performance.

## Data availability statement

The data used in the current research is not open-access. However, researchers can apply to access the dataset via Ungdata.no website. Further inquiries can be directed to the corresponding author.

## Ethics statement

The studies involving humans were approved by Ethical committee at the Department of Psychology, University of Oslo. The studies were conducted in accordance with the local legislation and institutional requirements. Informed consent for participation in this study was provided by the participants.

## Author contributions

EFH conceptualized the study and the research methods, carried out the data cleaning, missing data imputation, data analysis and visualization, as well as literature review and writing of the draft. NC and TVS were engaged in all steps of the study and provided input regarding multiple theoretical, technical, practical challenges, provided feedback on several drafts, and helped with revising the manuscript. All authors approved the final version of the manuscript for submission.
